# Analysis and prediction of nutritional outcome of patients with pediatric inflammatory bowel disease from Bahrain

**DOI:** 10.1186/s12887-024-04720-3

**Published:** 2024-04-24

**Authors:** Hasan M. Isa, Masooma Mohamed, Ahmed Alsaei, Zahra Isa, Enjy Khedr, Afaf Mohamed, Haitham Jahrami

**Affiliations:** 1 Pediatric Department, Salmaniya Medical Complex, Manama, Bahrain; 2 College of Medicine and Medical Science, Arabian Gulf University, Manama, Bahrain; 3 Faculty of Medicine, Mansoura University, Mansoura, Egypt; 4 Faculty of Medicine, Benha University, Benha, Egypt; 5 Ministry of Health, Manama, Bahrain

**Keywords:** Pediatric, Inflammatory bowel disease, Nutritional status, Overweight, Thinness

## Abstract

**Background:**

Inflammatory bowel disease (IBD) is a chronic gastrointestinal disease that causes anorexia, malabsorption, and increased energy requirements. Childhood IBD can significantly impact nutritional status and future health.

**Objective:**

This study aimed to analyze the nutritional status of patients with pediatric IBD at presentation and during follow-up and to identify predictors of nutritional outcome.

**Methods:**

This retrospective cohort study reviewed the medical records of children diagnosed with IBD in the Pediatric Department, Salmaniya Medical Complex, Bahrain, 1984 − 2023. Demographic data, clinical characteristics, and anthropometric data were collected. World Health Organization growth standards were used to interpret nutritional status.

**Results:**

Of the 165 patients, 99 (60%) had anthropometric data at presentation, and 130 (78.8%) had follow-up data. Most patients were males (64.6%) and had Crohn’s disease (CD) (56.2%), while 43.8% had ulcerative colitis (UC). The median age at presentation was 10.9 years and the mean follow-up duration was 12.6 years. At presentation, 53.5% of the patients were malnourished, that decreased to 46.9% on follow-up. Thinness was reduced from 27.3% at presentation to 12.1% at follow-up (*p* = 0.003). There was an increased tendency to normal weight on follow-up (59.6%) compared to time of presentation (46.5%), *p* = 0.035. Overweightness showed a non-significant increase from 26.3% at presentation to 28.3% at follow-up (*p* = 0.791). Children with IBD were more likely to become obese when they grow up to adulthood (2.3% versus 20.5%, respectively, *p* < 0.001). Weight-for-age, and height-for-age at presentation were higher among CD compared to UC, but body mass index (BMI) at follow-up was higher among UC patients (*p* < 0.05). Thinness at follow up was associated with very early-onset disease (*p* = 0.02), lower weight and BMI at presentation (*p* < 0.001 each), younger age at follow-up (*p* = 0.002), pediatric age group (*p* = 0.023), lower hematocrit (*p* = 0.017), and higher C-reactive protein (*p* = 0.007). Overweight at follow up was associated with increased weight and BMI at presentation (*p* < 0.001 each), longer disease duration (*p* = 0.005), older age (*p* = 0.002), and azathioprine intake (*p* = 0.026). Considering follow-up duration, univariate analysis exhibited that Bahraini nationality, post-diagnosis disease duration, age at follow-up, occurrence of diarrhea, height, and BMI at presentation were factors that decreased liability to abnormal nutritional status, while CD, history of weight loss, perianal disease, and skin rash, and intake of prednisolone expressed increased liability of abnormal nutritional status (*p* < 0.05).

**Conclusion:**

Pediatric IBD is associated with a high incidence of malnutrition. Thinness is more prominent at presentation, while overweight is higher on follow-up. Multiple risk factors aggravating abnormal nutritional status were highlighted. Accordingly, nutritional counseling should be prioritized in a multidisciplinary approach.

**Supplementary Information:**

The online version contains supplementary material available at 10.1186/s12887-024-04720-3.

## Introduction

Inflammatory bowel disease (IBD) includes Crohn’s disease (CD), ulcerative colitis (UC) and indeterminate colitis [1, 2]. The cause of IBD is unclear, but an immune reaction that is triggered by environmental factors in genetically susceptible individuals is the most accepted theory [1]. IBDs are chronic relapsing diseases affecting mainly the gastrointestinal tract [1, 2]. All these diseases can present at any age even during infancy [1, 3]. Patients presenting in infancy are more likely to develop complications [1]. Impairment of nutritional status and growth failure are important complications of childhood IBD [4 − 11]. A substantial number of children with IBD may suffer from malnutrition ranging from 20 to 85% [4]. Poor weight gain and weight loss are common at the time of IBD diagnosis in children and adolescents [3]. Children with early-onset IBD before puberty are usually thin due to slower growth [3, 5]. This can have a significant impact on the status of disease and the quality of life, future health, and well-being of these patients [6].

Several causes of undernutrition and growth failure exist in IBD patients [2, 7]. Inadequate intake, anorexia, increased gastrointestinal losses, malabsorption, increased energy requirements and oxidative stresses may play a role in malnutrition [8, 9]. Patients with CD are more prone to malnutrition and growth failure than are those with UC [6, 10, 11]. This difference might be attributed to the presence of transmural gastrointestinal inflammation in CD patients compared to the limited colonic mucosa inflammation in UC patients [11].

The main goal of managing children with IBD is to control the disease and promote growth [10]. Identification of malnutrition via nutritional assessment and treatment of nutritional deficits are needed [2]. A detailed initial assessment of nutritional status is crucial for planning appropriate management, reversing growth impairment, and improving health outcomes. This needs to be complemented by a proper continuous assessment [6, 9].

Data on the nutritional status of patients with pediatric IBD from developing countries are scarce [12, 13]. To date, there are no published studies about the nutritional status of pediatric IBD patients from Bahrain. Accordingly, this study aimed to analyze the nutritional status of patients with pediatric IBD at the time of diagnosis and at the last follow-up and to identify the predictors of nutritional outcome, including thinness and overweight.

## Methods

### Study design, setting, and population

In this retrospective cohort study, we reviewed the electronic medical records of patients with pediatric IBD diagnosed at the Department of Pediatrics, Salmaniya Medical Complex (SMC), Bahrain, between January 1984 and July 2023. SMC is the main tertiary hospital in Bahrain, and it is the only center that provides diagnostic and therapeutic services for pediatric patients with IBD. All patients with pediatric IBD and available anthropometric parameters were included in this study. Patients with no anthropometric data were excluded. The IBD diagnosis was based on the criteria of the IBD Working Group of the European Society of Pediatric Gastroenterology, Hepatology and Nutrition (ESPGHAN) and the North American Society of Pediatric Gastroenterology, Hepatology and Nutrition (NASPGHAN) [14, 15]. At SMC, four dedicated pediatric dietitians are assigned to run inpatient and outpatient pediatric nutrition services. Five outpatient dietetic clinics are available, one daily general pediatric nutrition clinic and four specialized multidisciplinary clinics [a pediatric weight loss clinic (once a week), a diabetes clinic (once a week), a diabetes insulin pump clinic (twice a week), a cystic fibrosis clinic (twice a month), and an oncology clinic (once a week)].

### Data collection

Data about sex, nationality, date of birth, type of delivery, birth weight, type of IBD, age at presentation, pre- and postdiagnosis duration of the illness, age at the time of anthropometric measurement, initial clinical presentations, extraintestinal manifestations, inflammatory markers such as erythrocyte sedimentation rate (ESR) and C-reactive protein (CRP), disease activity, and medical therapies used at the time of diagnosis or during the follow-up course were collected.

Patient anthropometric parameters were recorded at the initial presentation and during the last hospital admission or during the last visit to the outpatient clinic. Weight and height were measured using a calibrated Seca digital column scale with a stadiometer (Hamburg, Germany). The World Health Organization (WHO) ^“^WHO AnthroPlus” anthropometric software program version 3.2.2 (WHO, Geneva, Switzerland, 2011) was used to calculate the patient’s growth parameters at presentation and at the last follow-up. Weight for age in kilograms (kg), weight for age percentile (WAP), weight for age z score (WAZ), height for age in centimeters (cm), height for age percentile (HAP), height for age z score (HAZ), body mass index (BMI) [BMI = weight (kg)/height(m)^2^], BMI percentile (BMIp) and BMI z score (BMIz) were calculated. The growth parameters are presented as the standard deviation (SD) from age- and sex-specific reference means. WHO child growth standards from birth to 5 years of age, WHO growth references for school-age children and adolescents aged 5–19 years and the WHO adult BMI reference table were used as references for interpretation of nutritional status [16, 17]. Accordingly, in patients from birth to 19 years of age, thinness and severe thinness were defined as BMIz <-2 SD and <-3 SD, respectively, while possible risk of overweight, overweight and obesity were defined as BMIz for age > + 1, >+2 and > + 3 SD, respectively. In pediatric patients who became adults at follow up (older than 18 years), thinness was defined as BMI < 18.5 kg/m^2^, normal as BMI 18.5–24.9 kg/m^2^, overweight as BMI 25–29.9 kg/m^2^, and obesity as BMI > 30 kg/m^2^. Obesity was further classified into obesity class I (BMI = 30–34.9 kg/m^2^), class II (BMI = 35–39.9 kg/m^2^) and class III (BMI > 40 kg/m^2^). Stunting and severe stunting were defined as HAZ <-2 SD and <-3 SD, respectively.

### Ethics

This study was conducted in accordance with the principles of Helsinki Declaration, and it was ethically reviewed and approved by the Research Ethics Committee (REC) of the Ministry of Health, Kingdom of Bahrain (Approval number: 76,120,521, May 12, 2021). This study is by the relevant guidelines and regulations. The data in this study were obtained from this patient and his legal guardian. Written informed consent was obtained from the patient’s parents. Informed consent was obtained from the patient’s parents and/or legal guardians for the publication of identifying information/images in online open access publications.

### Statistical analysis

Patient data were analyzed using the Statistical Package for Social Sciences (SPSS) version 27. (SPSS, Inc., Chicago, USA), and GraphPad Prism, version 9.4.1 (GraphPad, San Diego, USA). Frequencies and percentages were calculated for categorical variables. Continuous variables are presented as the mean ± standard deviation (SD) for normally distributed variables or median and interquartile range (IQR) for nonnormally distributed variables. A test of normality was performed using the Kolmogorov‒Smirnov test.

Patient characteristics were compared based on the type of IBD (CD or UC) and nutritional status using bivariate analysis. Pearson’s *χ*^*2*^ test and Fisher’s exact test were used to compare categorical variables between groups. McNemar’s test was used to compare BMI nutritional status between the time of presentation and at follow-up. For comparisons between two or more groups for normally distributed variables, Student’s *t* test, paired-samples *t* test and one-way ANOVA were used, while the Mann‒Whitney U test and Kruskal‒Wallis’s test were used for nonnormally distributed variables.

Cox regression models were used to obtain hazard ratios (HRs). Multinominal logistic regression was also used to model the associations between the three levels of nutritional status (thinness, normal and overweight) and patient demographic data, the IBD disease activity, growth parameters, laboratory markers and medical therapy. The ordinal regression system was not used because the test of the parallel line was significant; therefore, to overcome this problem, multinomial regression was used instead. For the calculation of the predicted probabilities using multinomial logistic regression, the following equations were used:

$$\pi 1=\frac{\text{exp}\left({\alpha }_{1}+ {\beta }_{1}\chi \right)}{1+\text{exp}\left({\alpha }_{1}+ {\beta }_{1}{\chi }\right)+\text{exp}\left({\alpha }_{2}+ {\beta }_{2}\chi \right)}$$,

$$\pi 2=\frac{\text{exp}\left({\alpha }_{2}+ {\beta }_{2}\chi \right)}{1+\text{exp}\left({\alpha }_{1}+ {\beta }_{1}{\chi }\right)+\text{exp}\left({\alpha }_{2}+ {\beta }_{2}\chi \right)}$$

$$\pi 3=\frac{1}{1+\text{exp}\left({\alpha }_{1}+ {\beta }_{1}{\chi }\right)+\text{exp}\left({\alpha }_{2}+ {\beta }_{2}\chi \right)}$$

Odds ratios (ORs) were used to assess the association between the dependent and independent variables, and the confidence interval (CI) was set at 95%. A *P* value < 0.05 was considered to indicate statistical significance. A Kaplan‒Meier curve was also used to compare the nutritional status of the patients in relation to follow-up time.

## Results

During the study period, out of 165 patients diagnosed with pediatric IBD, 99 (60%) had anthropometric data available at presentation, and 130 (78.8%) had data available at follow-up; these patients were included in the study. The remaining 35 (21.2%) patients with unavailable anthropometric data were excluded. The demographic data and clinical characteristics of the study population are shown in Table 1. Most patients were males (*n* = 84, 64.6%), the majority were Bahraini (*n* = 112, 86.2%), and 18 (13.8%) were non-Bahraini [five (3.8%) patients were from India, four (3.1%) from Syria, three (2.3%) from Egypt, two (1.5%) from Pakistan, and one (0.8%) from Lebanon, Morocco, Qatar, and Switzerland each]. Seventy-three (56.2%) patients had CD, 57 (43.8%) had UC, and none had indeterminate colitis. Patients with UC presented with more diarrhea (*p* < 0.001), which is the most common symptom (*n* = 86, 71.7%), while those with CD presented later than did those with UC (*p* = 0.034), with more recurrent abdominal pain (*p* = 0.024), weight loss (*p* = 0.01) and perianal disease (*p* < 0.001). Patients with CDs had higher CRP levels (*p* < 0.001) than did those with UC. The median pediatric CD activity index was 32.5 (IQR: 25 − 42.5), and the mean pediatric UC activity index was 55.5 ± 15.1. Patients with UC had greater disease activity at presentation than did those with CD (*p* < 0.001).

**Table 1:** Demographic and clinical characteristics of patients with pediatric inflammatory bowel disease

Variables	IBD, *n* = 130	CD, *n* = 73 (56.2)	UC, *n* = 57 (43.8)	*p* value
**Demographic data**				
Sex				0.096^*^
Male	84 (64.6)	52 (71.2)	32 (56.1)	
Female	46 (35.4)	21 (28.8)	25 (43.9)	
Nationality				1.000^*^
Bahraini	112 (86.2)	63 (86.3)	49 (86.0)	
Non-Bahraini	18 (13.8)	10 (13.7)	8 (14.0)	
Age at presentation (yr)	10.9 (9.1–13)	11.4 (9.7–13)	10 (7.5–12.7)	**0.034** ^**†**^
IBD onset (Paris Classification)				**0.035** ^**‡**^
Infantile (< 2 year)	1 (0.8)	0 (0.0)	1 (1.8)	
Very-early onset (2 to < 6 year)	15 (11.5)	4 (5.5)	11 (19.3)	
Early onset (6 to < 10 year)	34 (26.2)	18 (24.7)	16 (28.1)	
Late onset (10 to < 18 year)	80 (61.5)	51 (69.9)	29 (50.9)	
Post-diagnosis disease duration (yr)	4.8 (1.3–11.1)	3.2 (1.2–8.7)	7.6 (2.5–13.7)	**0.017** ^**†**^
**Clinical presentations**	120 (92.3)	67 (91.8)	53 (93)	1.000^*^
Diarrhea	86 (71.7)	40 (59.7)	46 (86.8)	**0.001** ^*^
Bloody diarrhea	75 (62.5)	28 (41.8)	47 (88.7)	**< 0.001** ^*^
Recurrent abdominal pain	75 (62.5)	48 (71.6)	27 (50.9)	**0.024** ^*^
Weight loss	60 (50.0)	41 (61.2)	19 (35.8)	**0.010** ^*^
Pallor	49 (40.8)	24 (35.8)	25 (47.2)	0.262^*^
Anorexia	46 (38.3)	29 (43.3)	17 (32.1)	0.258^*^
Vomiting	36 (30.0)	21 (31.3)	15 (28.3)	0.841^*^
Arthralgia	30 (25.0)	16 (23.9)	14 (26.4)	0.833^*^
Perianal disease	28 (23.3)	26 (38.8)	2 (3.8)	**< 0.001** ^*^
Fever	22 (18.3)	13 (19.4)	9 (17)	0.815^*^
Constipation	18 (15.0)	14 (20.9)	4 (7.5)	0.061^*^
Skin rash	12 (10.0)	8 (11.9)	5 (9.4)	0.772^*^
Jaundice	6.0 (5.0)	2 (3.0)	4 (7.5)	0.404^*^
Hematemesis	5 (4.2)	2 (3.0)	3 (5.7)	0.654^*^
Extraintestinal manifestations^a^	47 (36.2)	24 (35.3)	18 (34.0)	0.852^*^
**Laboratory markers**				
Hematocrit (%), (*n* = 127)	30.9 (26.4–36)	30.9 (25.8–37.2)	30.3 (26.6–35.7)	0.553^**†**^
ESR (mm/h), (*n* = 120)	25 (15-44.8)	27 (15–46)	20 (13–44)	0.434^†^
CRP (mg/dL), (*n* = 118)	13.9 (1.6–48.2)	34.5 (7.4–65)	3.6 (1.0-17.5)	**< 0.001** ^**†**^
**Disease activity at presentation (** *n* ** = 121)**				**< 0.001** ^**‡**^
Mild	30 (24.8)	26 (38.2)	4 (7.5)	
Moderate	45 (37.2)	17 (25.0)	28 (52.8)	
Severe	46 (38)	36.8 (36.8)	21 (39.6)	
**Medications used**	119 (91.5)	65 (89%)	54 (94.7)	0.346^*^
Prednisolone	94 (79.0)	54 (83.1)	40 (74.1)	0.263^*^
Omeprazole	86 (72.3)	45 (69.2)	41 (75.9)	0.538^*^
Azathioprine	85 (71.4)	54 (83.1)	31 (57.4)	**0.002** ^*^
Folic acid	85 (71.4)	46 (70.8)	31 (57.4)	1.000^*^
Mesalazine	73 (61.3)	31 (47.7)	42 (77.8)	**0.001** ^*^
Iron supplementation	71 (59.7)	35 (53.8)	36 (66.7)	0.190^*^
Vitamin D supplementation	55 (46.2)	34 (52.3)	21 (38.9)	0.196^*^
Biological therapy	55 (46.2)	38 (58.5)	17 (31.5)	**0.005** ^*^
Calcium supplementation	42 (35.3)	25 (38.5)	17 (31.5)	0.448^*^
Multivitamins	37 (31.1)	21 (32.3)	16 (29.6)	0.843^*^
H2 blockers	6.0 (5.0)	1 (1.5)	5 (9.3)	0.090^*^
Ursodeoxycholic acid	5.0 (4.2)	0 (0.0)	5 (9.3)	**0.017** ^*^
Fat soluble vitamins (ADEK)	4.0 (3.4)	0 (0.0)	4 (7.4)	**0.040** ^*^
Vitamin B12 supplementation	3.0 (2.5)	0 (0.0)	3 (5.6)	0.091^*^
Other medications^b^	11 (9.2)	7 (9.6)	4 (7.0)	0.752^*^
Exclusive enteral nutrition	8 (6.2)	6 (8.2)	2 (3.5)	0.465^*^

Values are presented as number (%) or median (interquartile range). IBD, inflammatory bowel disease; CD, Crohn’s disease; UC, ulcerative colitis; ESR, erythrocytes sedimentation rate; CRP, C-reactive protein. ^a^Arthralgia (*n* = 30, 25%; four of them had arthritis), erythema nodosum (*n* = 12, 10%), conjunctivitis (*n* = 6, 4.6%), aphthous ulcers (*n* = 5, 3.8%), sclerosing cholangitis, overlap syndrome, autoimmune hemolytic anemia, thromboembolic manifestation (*n* = 2, 1.5% each), while autoimmune hepatitis, nephritis, clubbing, ankylosis spondylitis and gall bladder edema (*n* = 1, 0.8% each). Some patients had more than one extraintestinal manifestation. ^b^Somatotropin (*n* = 4), methotrexate (*n* = 2), testosterone, tacrolimus, colchicine, loperamide hydrochloride, isoniazid with vitamin B6 for latent tuberculosis prophylaxis, lactulose, and hydroxyurea for sickle cell disease (*n* = 1 each). Some patients received more than one medication. ^*^Fisher’s exact test, ^†^Mann-Whitney *U* test, ^‡^Pearson’s *χ*^*2*^ test. Boldface indicates a statistically significant difference with *p* < 0.05.

The most frequent medications used was prednisolone (*n* = 94, 79%). Biological therapy was used in 55 (46.2%) patients [adalimumab alone in 38 (31.9%) patients, infliximab alone in 12 (10.1%), both adalimumab and infliximab in four (3.4%), and one (0.8%) patient received an unspecified type of biological therapy]. Two (1.7%) patients received ustekinumab after failure of infliximab therapy. Patients with CD used more azathioprine (*p* = 0.002) and more biological therapy (*p* = 0.005), specifically adalimumab [26 (40%) versus 12 (22.2%), *p* = 0.049], while those with UC used more mesalazine (*p* = 0.001). Patients with UC had a longer postdiagnosis disease duration (*p* = 0.017).

The basic anthropometric data of the patients at presentation and at follow-up are shown in Table 2. At presentation, 99 (76.2%) patients had anthropometric data available, while all 130 patients had available follow-up data. Patients with UC had a lower median weight [27.6 (IQR: 20.5 − 38.0) versus 33.5 (IQR: 24.5 − 46.0) kg] and mean height [133.6 ± 21.3 versus 146.4 ± 16.3 cm] at presentation than did those with CD (*p* = 0.023 and *p* < 0.001, respectively), but there was no significant difference in BMI or BMIz.

**Table 2:** Basic anthropometric data of patients with pediatric inflammatory bowel disease

Anthropometric parameters	IBD, *n* = 130	CD, *n* = 73	UC, *n* = 57	*p* value (95% CI)
**Birth weight (kg), **(*n*** = 57)**	3.0 (2.9–3.5)	3.0 (2.9–3.4)	3.0 (2.9–3.5)	0.697^*^
**Age at presentation (yr)**	10.9 (9.1–13)	11.4 (9.7–13)	10 (7.5–12.7)	**0.034** ^**†**^
**Growth parameters at presentation (** *n* ** = 99)**				
BMI z-score	-0.5 ± 2.2	-0.8 ± 2.4	0.03 ± 1.9	0.054^†^ (-1.8-0.02)
BMI z-score group				0.455^§^
<-3 SD	12 (12.1)	11 (18.0)	1 (2.6)	
<-2 SD	15 (15.2)	9 (14.8)	6 (15.8)	
<-1 SD	11 (11.1)	7 (11.5)	4 (10.5)	
0 SD	35 (35.4)	20 (32.8)	15 (39.5)	
>+1 SD	10 (10.1)	5 (8.2)	5 (13.2)	
>+2 SD	11 (11.1)	6 (9.8)	5 (13.2	
>+3 SD	5 (5.1)	3 (4.9)	2 (5.3)	
**Age at last growth assessment (yr) (** *n* ** = 130)**	14.2 (12.6–23.2)	14.0 (12.8–20.4)	15.1 (12-26.8)	0.334^*^
**Age group at last growth assessment (yr)**				0.353^‡^
Pediatric (0–18 year)	86 (66.1)	51 (69.9)	35 (61.4)	
Adult (> 18 year)	44 (33.9)	22 (30.1)	22 (38.6)	
**Growth parameters on follow up (** *n* ** = 130)**				
**Patients from 0 to 19 years (** *n* ** = 87, 66.9%)**				
WAP	31 (6–69)	21 (4–53)	52.5 (10.5–76.7)	**0.041** ^*^
WAZ	-0.6 ± 1.6	-0.8 ± 1.6	-0.1 ± 1.4	**0.040** ^**†**^ **(-1.4- -0.03)**
HAP	22 (6.3–51.3)	22 (2.2–51.3)	21.1 (10-52.9)	0.372^*^
HAZ	-0.8 (-1.5-0.03)	-0.8 (-2.0-0.0)	-0.8 (-1.3-0.1)	0.361^*^
BMI percentile	31.5 (6.7–87)	27.4 (3.6–80.3)	64.2 (14.3–90.8)	0.079^*^
BMI z-score	-0.3 ± 1.8	-0.5 ± 1.9	0.1 ± 1.7	0.127^†^ (-1.4-0.2)
BMI z-score group				0.487^§^
<-3 SD	5 (5.7)	4 (7.8)	1 (2.8)	
<-2 SD	8 (9.2)	5 (9.8)	3 (8.3)	
<-1 SD	17 (19.5)	11 (21.6)	6 (16.7)	
0 SD	33 (37.9)	19 (37.3)	14 (38.9)	
>+1 SD	15 (17.2)	8 (15.7)	7 (17.4)	
>+2 SD	7 (8.0)	2 (3.9)	5 (13.9)	
>+3 SD	2 (2.3)	2 (3.9)	0 (0.0)	
**Patients older than 19 years (** *n* ** = 43, 33.1%)**				
BMI (kg/m^2^)	25.9 ± 6.3	24.3 ± 5.8	27.5 ± 6.6	0.098^†^ (-7.0-0.6)
BMI group (kg/m^2^)				0.305^§^
<18.5	2 (4.7)	1 (4.5)	1 (4.8)	
18.5–24.9	19 (44.2)	13 (59.1)	6 (28.6)	
25-29.9	13 (7.0)	4 (18.2)	9 (42.9)	
30-34.9	5 (11.6)	3 (13.6)	2 (9.5)	
35-39.9	3 (7.0)	1 (4.5)	2 (9.5)	
>40	1 (2.3)	0 (0.0)	1 (4.8)	

Values are presented as number (%), mean ± standard deviation, or median (interquartile range). IBD, inflammatory bowel disease; CD, Crohn’s disease; UC, ulcerative colitis; CI, confidence interval; BMI, body mass index; WAP, weight-for-age percentile; WAZ, weight-for-age z-score; HAP, height-for-age percentile; HAZ, height-for-age z-score; SD, standard deviation. ^*^Mann-Whitney *U* test, ^†^Student’s *t* test, ^‡^Fisher’s exact test, ^§^Pearson’s *χ*^*2*^ test. Boldface indicates a statistically significant difference with *p* < 0.05.

The median age at the time of the last growth assessment was 14.2 (IQR: 12.6 − 23.2) years; 87 (66.9%) patients were ≤ 19 years, while 43 (33.1%) patients were older than 19 years. Patients with CD were more likely to be aged 10-14.9 years (*n* = 46, 63%), while patients with UC were more likely to be aged > 18 years (*n* = 22, 38.6%) (*p* < 0.001); however, there was no significant difference between pediatric patients and those who reached adulthood (*p* = 0.353). Patients with UC had a higher median BMI than did those with CD at the last growth measurement [22.2 (IQR: 17.5 − 27.1) versus 19.0 (IQR: 16.3 − 24.6) kg/m^2^, respectively, *p* = 0.032]. In the 0 to 19 years old group, patients with UC had significantly greater WAP and WAZ than those with CD (*p* = 0.041 and 0.040, respectively). Among patients older than 19 years, patients with UC had higher BMIs at the time of the last growth assessment, but the difference was not significant.

According to the follow-up data of the 99 patients with available BMIs at presentation, the mean BMI at presentation was lower than that at the last follow-up [18.3 ± 11.0 versus 19.7 ± 5.2 kg/m^2^], but these differences were not significant (*p* = 0.205; 95%CI -3.7 − 0.8).

**Fig. 1:**
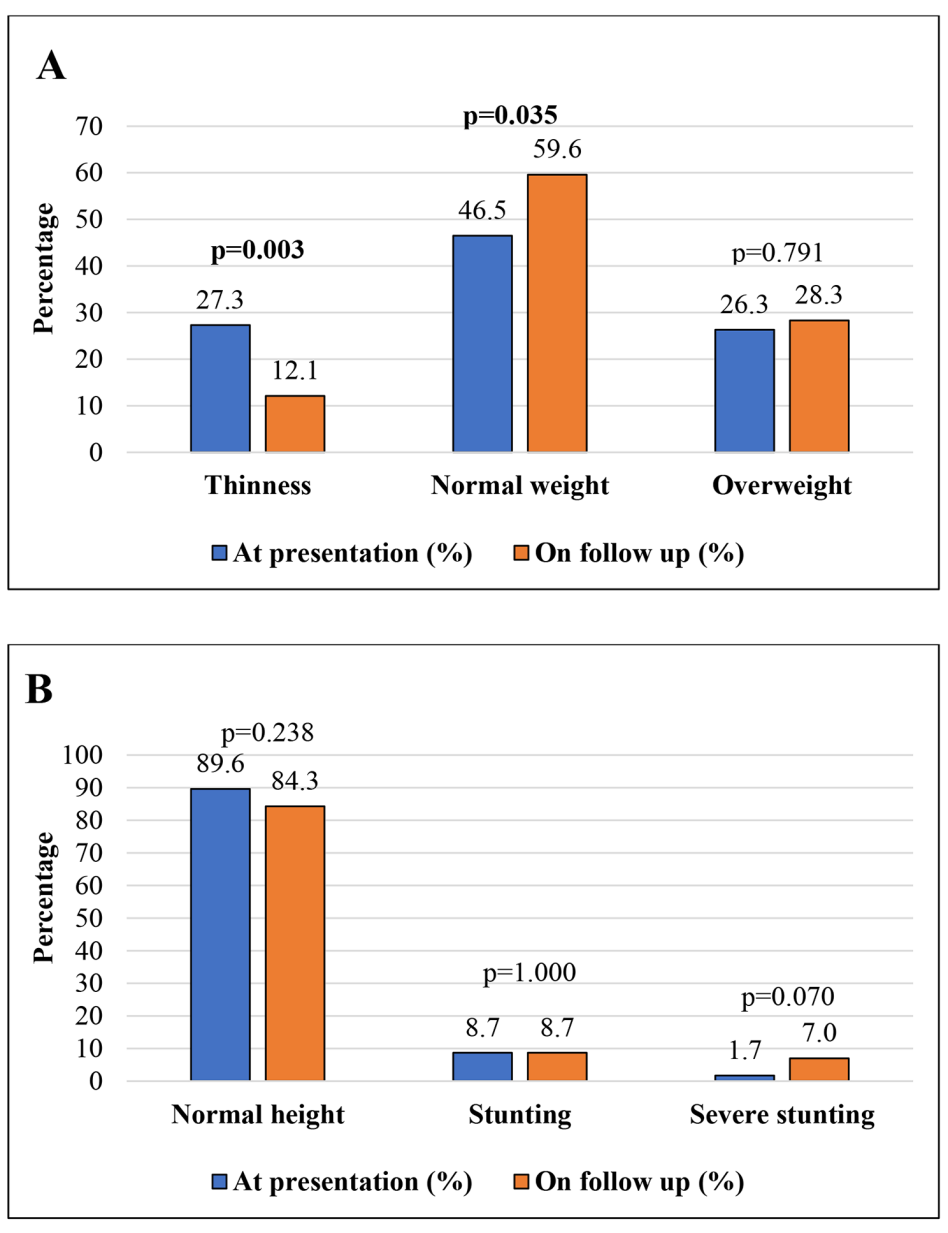
Nutritional status **(A)** and linear growth **(B)** of patients with pediatric inflammatory bowel disease at presentation and on follow-up (*n* = 99). *P* value was calculated using McNemar’s test (Boldface indicates a statistically significant difference with *p* < 0.05)

The results of the nutritional assessments are shown in Fig. 1. At presentation, 46/99 (46.5%) patients had a normal nutritional status, while 53/99 (53.5%) were malnourished. Thinness was noted in 27 (27.3%) patients [15 (15.2%) patients were moderately thin (BMI <-2 SD), and 12 (12.1%) were severely thin (BMI <-3 SD)]. Twenty-six (26.3%) patients had weights higher than normal [possible risk of overweight (BMI > + 1 SD) in 10 (10.1%), overweight (BMI > + 2 SD) in 11 (11.1%), and obesity (BMI > + 3 SD) in five (5.1%) patients]. Upon follow-up of the 99 patients with available anthropometric data at presentation, there was a significant reduction in thinness (*p* = 0.003) and an increase in normal nutritional status (*p* = 0.035). Of the 27 (27.3%) patients with thinness at presentation, eight (29.6%) remained thin at follow-up, 17 (63%) became normal, and two (7.4%) became overweight. Of the 46 (46.5%) patients with a normal nutritional status, 36 (78.3%) remained normal, four (8.7%) became thin, and six (13%) became overweight. Of the 26 (26.3%) overweight patients at presentation, 20 (76.9%) remained overweight, while six (23.1%) became normal at follow-up. At presentation, stunting was detected in 12 (12.1%) patients, which increased to 18 (18.2%) patients at follow-up; however, this difference was not significant (*p* = 0.238).

At the time of the last growth assessment, 69 (53.1%) patients had a normal nutritional status, and 61 (46.9%) were malnourished. Thinness was found in 15 (11.5%) patients [10 (7.7%) were moderately thin, and five (3.8%) were severely thin]. Forty-six (35.4%) patients had a weight higher than normal [14 (10.8%) had a possible risk of overweight, 19 (16.2%) were overweight and 11 (8.5%) were obese]. Obesity class I was noted in six (4.6%) patients, class II in four (3.1%) patients and class III in one (0.8%) patient. Obesity was noted in two out of 86 (2.3%) pediatric patients, and nine (20.5%) out of 44 were adults (*p* < 0.001). Stunting was detected in 21 (16.2%) patients. Stunting was noted in 21 (16.2%) patients, 11 (52.4%) of them were severely stunted.

Bivariate analysis was performed to examine whether there was a statistically significant effect of the different studied variables on nutritional status (thinness, normal, and overweight), as shown in Table 3. Patients with Bahraini nationality, those born vaginally and those with non-very early onset IBD were more prone to having a normal nutritional status than were those without Bahraini nationality, those born via cesarean section, and those with very early-onset disease (*p* = 0.018, *p* = 0.030, and *p* = 0.020, respectively). The median postdiagnosis disease duration was significantly greater in the overweight group (9.2 years) than in the thin group (5.3 years), while the shortest duration was observed in the normal group (3.4 years) (*p* = 0.005).

**Table 3:** Bivariate analysis of the relationship of nutritional status on follow-up of patients with different studied variables

Variables	Nutritional status of 130 patients with IBD			*P* value
	Thinness, *n* = 15 (11.5)	Normal, *n* = 69 (53.1)	Overweight, *n* = 46 (35.4)	
**Sex**				0.854^*^
Male	6 (13.0)	25 (54.3)	15 (32.6)	
Female	9 (10.7)	44 (52.4)	31 (36.9)	
**Nationality**				**0.018** ^*^
Bahraini	15 (13.4)	54 (48.2)	43 (38.4)	
Non-Bahraini	0 (0.0)	15 (83.3)	3 (16.7)	
**Type of delivery, (** *n* ** = 54)**				**0.030** ^*^
Vaginal	8 (17.0)	28 (59.6)	11 (23.4)	
Caesarean	0.0 (0.0)	2.0 (28.6)	5.0 (71.4)	
**Birth weight (kg), (** *n* ** = 57)**	3.0 (2.8–3.3)	3.1 (3.0-3.4)	3.0 (2.7–3.7)	0.645^†^
**Age at presentation (yr)**	9.3 (4.9–12.3)	11.2 (9.1–12.7)	11.0 (9.6–13.6)	0.184^†^
**Age group at presentation (yr)**				**0.020** ^*^
Very early onset (< 6 year)	5 (31.3)	5 (31.5)	6 (37.5)	
Non-very early onset (6–18 year)	10 (8.8)	64 (56.1)	40 (35.1)	
**Type of IBD**				0.093^*^
Crohn’s disease	10 (13.7)	43 (58.9)	20 (27.4)	
Ulcerative colitis	5 (8.8)	26 (45.6)	26 (45.6)	
**Prediagnosis disease duration (mo), (** *n* ** = 65)**	1.0 (1.0–4.0)	1.3 (0.3-4.0)	3.0 (0.4-5.0)	0.784^†^
**Postdiagnosis disease duration (yr)**	5.3 (0.9–8.6)	3.4 (1.1–8.6)	9.2 (2.5–17)	**0.005** ^**†**^
**Extraintestinal manifestation, (** *n* ** = 120)**				0.229^*^
Yes	3 (6.4)	29 (61.7)	15 (31.9)	
No	12 (16.4)	37 (50.7)	24 (32.9)	
**Growth parameters at presentation**				
Weight (kg), (*n* = 95)	19.2 (15.8–27.8)	29.6 (23.0-34.7)	43.6 (27.8–52.0)	**< 0.001** ^**†**^
Height (cm), (*n* = 79)	128.1 ± 22.5	135.4 ± 17.0	142.2 ± 16.0	0.103^‡^
BMI (kg/m^2^), (*n* = 79)	13.2 (12.1–14.3)	15.4 (14.2–17.3)	21.7 (17.9–23.4)	**< 0.001** ^**†**^
**Age at last growth parameters (yr)**	13.3 (12.1–18.5)	13.8 (12.4–20.3)	16.9 (14.0-29.2)	**0.002** ^**†**^
**Age at last growth parameters (yr)**				**0.023** ^*^
≤18	13 (15.1)	49 (57.0)	24 (27.9)	
>18	2 (4.5)	20 (45.5)	22 (50.0)	
**Laboratory markers**				
Hematocrit (%), (*n* = 127)	25.8 (22.5–29.4)	31.5 (27–36)	31.7 (27.9–37.5)	**0.017** ^**†**^
ESR (mm/h), (*n* = 120)	30 (22–40)	26 (15–45)	20 (11–46)	0.274^†^
CRP (mg/dL), (*n* = 118)	43.6 (25.6–73.4)	14.6 (1.7–48.2)	4.7 (1.1–27.8)	**0.007** ^**†**^
**IBD disease activity, (** *n* ** = 121)**				0.831^*^
Mild	3 (10)	19 (63.3)	8 (26.7)	
Moderate	5 (11.1)	25 (55.6)	15 (33.3)	
Severe	7 (15.2)	23 (50.0)	16 (34.8)	
**Medication used, (** *n* ** = 119)**	15 (12.6)	63 (52.9)	41 (34.5)	
Prednisolone				0.158^*^
Yes	14 (14.9)	51 (54.3)	29 (30.9)	
No	1 (4.0)	12 (48.0)	12 (48.0)	
Azathioprine				**0.026** ^*^
Yes	12 (14.1)	38 (44.7)	35 (41.2)	
No	1 (2.9)	24 (70.6)	9 (26.5)	
Mesalazine				0.166^*^
Yes	11 (15.1)	35 (47.9)	27 (37.0)	
No	2 (4.3)	27 (58.7)	17 (37.0)	
Biological therapy				0.208^*^
Yes	10 (18.2)	26 (47.3)	19 (34.5)	
No	5 (7.8)	37 (57.8)	22 (34.4)	
Exclusive enteral nutrition				0.566^*^
Yes	0 (0.0)	5 (62.5)	3 (37.5)	
No	15 (12.3)	64 (52.5)	43 (35.2)	

Data presented as number (%) or mean ± standard deviation. IBD, inflammatory bowel disease; CD, Crohn’s disease; UC, ulcerative colitis; ESR, erythrocytes sedimentation rate; CRP, C-reactive protein. ^*^Pearson’s *χ*^*2*^ test, ^†^Kruskal Wallis’s test, ^‡^One way ANOVA. Boldface indicates a statistically significant difference with *p* < 0.05.

The thinness group had a lower weight and BMI at presentation (*p* < 0.001 each), was youngest at the time of last growth measurement (*p* = 0.002), was more common in the pediatric age group (*p* = 0.023), had a lower hematocrit percentage (*p* = 0.017), and had a higher median CRP level (*p* = 0.007) than did the other groups. Compared with other nutritional groups, overweight was associated with greater weight and BMI at presentation (*p* < 0.001 each), longer disease duration (*p* = 0.005), older age (*p* = 0.002), and greater azathioprine intake (*p* = 0.026). Although there was no significant difference in the three types of nutritional status based on IBD type (*p* = 0.093), patients with UC were more likely to be in the overweight group (*n* = 26, 45.6%) than were those with CD (*n* = 20, 27.4%) (*p* = 0.042). Other factors, such as sex, birth weight, age at presentation, prediagnosis disease duration, presence of extraintestinal manifestations, height at presentation, ESR level, and the use of mesalazine, biologic therapy, and exclusive enteral nutrition, were not significantly different among the three nutritional groups.

Considering follow-up duration, univariate analysis exhibited that Bahraini nationality, post-diagnosis disease duration, age at follow-up, occurrence of diarrhea, height, and BMI at presentation were factors that decreased liability to abnormal nutritional status, while CD, history of weight loss, perianal disease, and skin rash, and intake of prednisolone expressed increased liability of abnormal nutritional status (*p* < 0.05) (Table 4). The hazard ratio (HR) for abnormal nutritional status was 0.3 (95%CI 0.2 − 0.5) compared with normal nutritional status subjects. Individuals of the Bahraini ethnicity were 3.98 times more prone to overweight than participants with a normal nutritional status were (38.4% probability of being overweight) (Supplementary Table 2). Normal delivery participants were less likely to develop overweight (OR = 0.16, 95%CI 0.03 − 0.93), with a predicted probability of 23.4%. According to the Cox regression, patients with CD were 1.95 times more likely to develop abnormal nutritional status, while according to the multinominal logistic regression, these patients were less likely to develop overweight (OR = 0.47, 95%CI 0.22 − 0.99). Only 27.4% of patients with CD were likely to develop overweight.

**Table 4:** Univariate Cox and multinomial logistic regression for nutritional status of patients with pediatric inflammatory bowel disease

Variable	Cox regression		Multinomial logistic regression				
	Abnormal vs. normal		Thinness vs. normal		Overweight vs. normal		
	HR (95% CI)	*P* value	OR (95% CI)	*P* value	OR (95% CI)	*P* value	
**Demographic data**							
Male sex	1.07 (0.61–1.66)	0.979	0.85 (0.27–2.68)	0.784	1.17 (0.53–2.58)	0.690	
Bahraini nationality	0.3 (0.17–0.54)	**< 0.001**	58930879.04	1.000	3.98 (1.08–14.65)	**0.038**	
Normal delivery	0.46 (0.1–2.12)	0.316	22198171.35	1.000	0.16 (0.03–0.93)	**0.042**	
Crohn’s disease IBD type	1.95 (1.18–3.23)	**0.009**	1.21 (0.37–3.93)	0.752	0.47 (0.22–0.99)	**0.048**	
Pre-diagnosis disease duration	1.01 (0.96–1.06)	0.725	1.05 (0.98–1.11)	0.160	1.03 (0.97–1.09)	0.332	
Post-diagnosis disease duration	0.0 (0.0–0.0)	**< 0.001**	1.02 (0.93–1.12)	0.676	1.11 (1.04–1.17)	**< 0.001**	
Age at presentation	0.99 (0.93–1.05)	0.694	0.86 (0.74–1.01)	0.063	1.02 (0.91–1.13)	0.769	
Age at follow up	0.81 (0.77–0.86)	**< 0.001**	0.97 (0.89–1.06)	0.547	1.08 (1.03–1.13)	**0.002**	
Very early onset IBD	0.42 (0.17–1.05)	0.064	6.4 (1.57–26.15)	**0.010**	1.92 (0.55–6.71)	0.307	
**Clinical manifestation**							
Diarrhea	0.52 (0.31–0.89)	**0.016**	2.0 (0.51–7.83)	0.320	1.67 (0.68–4.12)	0.268	
Recurrent abdominal pain	1.09 (0.65–1.82)	0.746	0.57 (0.18–1.78)	0.334	0.8 (0.35–1.82)	0.595	
Weight loss	1.7 (1.03–2.8)	**0.037**	3.33 (0.86–12.92)	0.082	0.37 (0.16–0.85)	**0.020**	
Pallor	1.61 (0.97–2.67)	0.067	0.96 (0.31–3.02)	0.948	1.01 (0.45–2.25)	0.991	
Anorexia	1.3 (0.8–2.13)	0.296	0.96 (0.31–3.02)	0.948	0.72 (0.32–1.65)	0.441	
Vomiting	1.03 (0.61–1.74)	0.903	1.43 (0.45–4.54)	0.545	0.64 (0.26–1.59)	0.340	
Arthralgia	1.12 (0.64–1.94)	0.695	0.72 (0.18–2.87)	0.642	0.99 (0.4–2.46)	0.989	
Perianal disease	1.92 (1.06–3.45)	**0.031**	1.14 (0.32–4.07)	0.844	1.08 (0.43–2.69)	0.873	
Fever	1.5 (0.81–2.78)	0.198	0.63 (0.13–3.13)	0.570	0.89 (0.32.-2.47)	0.826	
Constipation	1.72 (0.89–3.32)	0.107	0.77 (0.15–3.9)	0.751	0.74 (0.24–2.3)	0.597	
Skin rash	2.43 (1.26–4.69)	**0.008**	0.0 (0.0–0.0)	1.000	0.27 (0.06–1.29)	0.101	
Jaundice	0.84 (0.31–2.32)	0.739	2.39 (0.39–14.42)	0.344	0.0 (0.0–0.0)	1.000	
Hematemesis	1.5 (0.47–4.82)	0.492	1.5 (0.15–15.51)	0.734	0.55 (0.06–5.51)	0.613	
Extraintestinal manifestations	1.44 (0.88–2.35)	0.147	0.32 (0.08–1.24)	0.098	0.8 (0.36–1.79)	0.583	
**Disease activity at presentation**							
Moderate	0.8 (0.43–1.48)	0.474	1.93 (0.44–8.49)	0.386	1.65 (0.58–4.69)	0.346	
Severe	0.81 (0.44–1.48)	0.490	1.27 (0.27–5.97)	0.765	1.43 (0.5–4.05)	0.507	
**Growth parameter**							
Birth weight	1.57 (0.67–3.71)	0.300	0.49 (0.13–1.85)	0.289	1.03 (0.3–3.52)	0.962	
Weight at presentation	0.99 (0.97-1.0)	0.129	0.91 (0.84–0.98)	**0.015**	1.06 (1.02–1.1)	**0.002**	
Height at presentation	0.99 (0.98–0.99)	**0.041**	0.97 (0.94-1.0)	0.054	1.02 (0.99–1.05)	0.070	
BMI at presentation	0.91 (0.86–0.98)	**0.009**	1.16 (1.04–1.29)	**0.006**	1.16 (1.04–1.28)	**0.005**	
**Laboratory test**							
Hematocrit %	0.99 (0.97–1.02)	0.684	0.94 (0.89–0.99)	**0.034**	1.0 (0.96–1.05)	0.895	
ESR	1.0 (0.99–1.02)	0.569	1.01 (0.98–1.04)	0.554	1.0 (0.98–1.02)	0.963	
CRP	1.0 (0.99–1.01)	0.571	1.01 (0.99–1.02)	0.128	0.99 (0.98–1.01)	0.367	
**Medical therapy**							
Prednisolone	1.94 (1.02–3.7)	**0.043**	3.29 (0.39–27.55)	0.271	0.57 (0.23–1.43)	0.230	
Azathioprine	0.94 (0.55–1.61)	0.823	115975454.6	1.000	0.89 (0.39–2.07)	0.799	
Mesalazine	0.95 (0.58–1.57)	0.848	0.8 (0.26–2.49)	0.704	1.51 (0.66–3.46)	0.326	
Biological therapy	0.66 (0.4–1.1)	0.109	2.85 (0.87–9.31)	0.084	1.23 (0.56–2.72)	0.610	
Exclusive enteral nutrition	1.48 (0.64–3.43)	0.360	1.62 (0.29–8.91)	0.582	0.0 (0.0–0.0)	1.000	
Omeprazole	1.14 (0.65-2.0)	0.640	2.4 (0.49–11.77)	0.280	0.71 (0.3–1.67)	0.436	
Folic acid	1.44 (0.83–2.51)	0.200	2.6 (0.53–12.7)	0.238	0.77 (0.33–1.8)	0.548	
Iron supplementation	1.36 (0.82–2.26)	0.237	1.5 (0.46–4.9)	0.502	1.17 (0.53–2.61)	0.698	
Vitamin D supplementation	1.29 (0.78–2.13)	0.323	1.88 (0.6–5.9)	0.282	0.98 (0.44–2.16)	0.957	
Calcium supplementation	0.68 (0.39–1.18)	0.171	3.09 (0.97–9.83)	0.056	1.92 (0.83–4.41)	0.126	
Multivitamins	1.17 (0.68–2.02)	0.576	3.47 (1.08–11.13)	**0.036**	0.65 (0.26–1.63)	0.358	
Ursodeoxycholic acid	0.98 (0.31–3.16)	0.978	1.43 (0.14–14.78)	0.765	0.5 (0.05–4.98)	0.554	
Fat soluble vitamins	0.77 (0.19–3.18)	0.722	2.18 (0.18–25.75)	0.537	0.76 (0.07–8.7)	0.827	
Other medications	0.35 (0.11–1.12)	0.076	5.0 (0.9-27.82)	0.066	2.78 (0.63–12.32)	0.179	

Boldface indicates a statistically significant difference with *P* < 0.05. HR: Hazard ratio, CI: confidence interval; OR: odd ratio; IBD: inflammatory bowel disease; BMI: body mass index; ESR: erythrocyte sedimentation rate; CRP: C-reactive protein.

There was a significant relationship between abnormal nutritional status and postdiagnosis disease duration (OR = 0.0, 95%CI 0.0 − 0.0) and age at follow-up (OR = 0.81, 95%CI 0.77 − 0.86). An increased postdiagnosis disease duration and age of follow-up were linked to a greater risk of overweight (*p* < 0.05). With a percentage of 53.5, patients with a mean postdiagnosis disease duration of 7.24 years were likely to be of normal weight; similarly, patients with a mean age at follow-up of 17.95 years were likely to have a normal weight of 54.4%. Moreover, very early-onset IBD was associated with an increased risk of thinness (OR = 6.4, 95%CI 1.57 − 26.15), with a predicted probability of 31.25%.

**Fig. 2:**
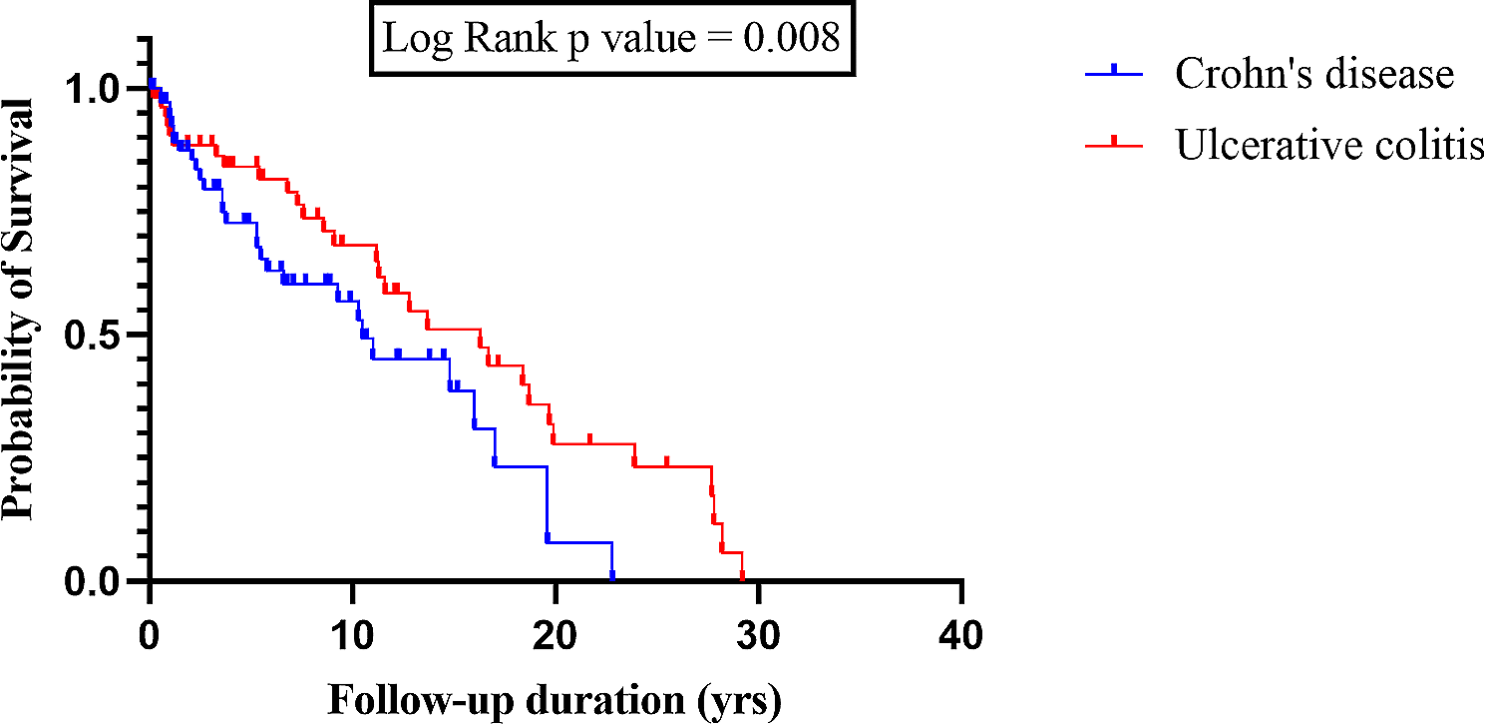
Kaplan-Meier survival curve for nutritional status of patients with pediatric inflammatory bowel disease

Kaplan-Meier curve estimated times to occurrence of abnormal nutritional status during the follow-up duration (Fig. 2). The mean follow-up duration until occurrence of abnormal nutritional status for all studied participants was 12.58 years (95%CI 10.31 − 14.85), which differs significantly between patients with CD and those with UC (*p* = 0.008). The mean follow-up duration until occurrence of abnormal nutritional status for patients with CD was 8.86 years (95%CI 6.7 − 11.0), which is much less than that of patients with UC with mean of 15.86 years (95%CI 1.75 − 12.42).

## Discussion

This study showed that pediatric IBD is associated with a high prevalence of malnutrition. While thinness was anticipated to be high at presentation, overweightness, or even obesity, was unexpectedly high both at presentation and at follow-up. At presentation, 27.3% of participants were thin which significantly decreased to 12.1% at follow-up (*p* = 0.003). This prevalence of underweight is greater than that of healthy children and adults from the general population. In Bahrain, the prevalence of underweight in children up to the age of five years, children aged 10 to 12 years, and adults was estimated to be 3.2%, 3.3% and 4%, respectively [18–20]. Aurangzeb et al. and Kuloglu et al. reported a comparable incidence rate of thinness among children with IBD of 14.3% and 14.9%, respectively [6, 21]. However, this incidence is considered low when compared to that reported in several pediatric and adult studies in which the prevalence of thinness ranged between 27 and 100% [8, 9, 11, 12, 22, 23]. Nutritional status in patients with IBD in neighboring countries and worldwide are shown in Supplementary Table 1 [5 − 8, 11, 14, 22 − 24]. Nonetheless, Kalantari et al. reported a lower prevalence of thinness among adult patients with UC of 9.1% (nine out of 99), which is similar to that of our study (8.8%) [4]. Thinness in patients with IBD is an expected finding due to the impact of the disease on patients’ oral nutrient intake, chronic gastrointestinal losses, and increased energy expenditure [8, 9]. These patients are usually anorexic secondary to abdominal pain and a sense of fullness, vital nutrient deficiencies, side effects of medications, disease complications such as strictures or abscesses, and psychological stresses. Moreover, children with IBD have lower levels of serum leptin than healthy controls [6].

In the present study, the prevalence of overweight was 26.3% at presentation, which further increased to 28.3% at the follow-up assessment. Although this rise was not statistically significant (*p* = 0.791), this prevalence is considered high when compared to that reported by El Mouzan et al. and Kuloglu et al., who reported a prevalence of 16% and 3.1% in children with IBD [12, 21]. Moreover, other studies, such as Aurangzeb et al. and Benjamin et al., reported a percentage of zero for overweight [6, 8]. This high prevalence of overweight in our study was an unexpected finding. However, this can be attributed to the general increase in the level of overweight among children and adults in Bahrain. In children up to five years of age, the prevalence of overweight and obesity was estimated to be 4.9% and 1.3%, respectively with a combined prevalence of 6.2% [18]. In children aged 10 to 12 years, this prevalence increased to 21.7% and 22.5%, respectively [19]. Ghareeb and Rasheed reported that Bahraini children were heavier than their counterparts 10 to 15 years ago [24]. Moreover, about one-third of the Bahraini adults are overweight and 42.8% are obese, while the corresponding percentages among non-Bahraini are 39.8% and 25.7% [20]. The high rate of overweight among children with and without IBD could be attributed to improved living conditions, improved economic status, sedentary lifestyles, and the westernization of dietary habits [12, 24]. Children and adolescents in Bahrain are shifting toward unhealthy dietary habits. They are consuming substantial amounts of high sugar-containing diet and high energy foods rich in saturated fat and dietary cholesterol in parallel with low intake of dietary fibers [25, 26]. This might increase their future risk of obesity and cardiovascular diseases. Accordingly, the importance of healthy balanced diet should be emphasized through implementation of tailored nutrition education programs in the schools to ensure normal growth and health maintenance of these children [25, 26]. Furthermore, in Bahrain, obesity reached the highest level among adults with the lowest educational levels (41.2%) and among those with the highest wealth quintiles [20]. Accordingly, physicians should be aware that overweight, rather than thinness, at presentation in patients with IBD is not an uncommon nutritional status [12].

The differences in the prevalence of malnutrition (thinness or overweight) between our cohort and patients from other countries can also be explained to some extent by differences in the methods used to assess nutritional status. While most of the reviewed studies used the WHO growth references for nutritional assessment, few studies used national growth charts or Centers for Disease Control and Prevention (CDC) references and classifications [6, 8, 9, 23]. In this study, WHO anthropometric definitions and references were used to assess nutritional status because Bahrain did not have a reference growth chart based on local anthropometric data. Using the WHO as a reference can aid in comparisons of the results of similar studies conducted in different countries [12, 13]. Nonetheless, WHO references might overestimate the prevalence of thinness when compared to national references and should be interpreted with caution [27].

This study investigated the possible risk factors for malnutrition in patients with pediatric IBD and showed no significant effect of sex on patient nutritional status. These findings were also described by El Mouzan et al. and Song et al. [9, 12]. However, Sentongo et al. reported a significantly lower weight for age z score in children and adolescent males with CD (*p* = 0.00002) than in females (*p* = 0.8) when compared to a control group [28]. In contrast, Benjamin et al. reported a greater incidence of malnutrition among adult females with CD than among males, which was explained by differences in dietary intake [8].

The present study showed that younger age, especially in the pediatric age group, was significantly associated with thinness, while older age was associated with overweight. El Mouzan et al. also showed that patient age has an impact on nutritional status. Children older than 10 years of age had a significantly greater incidence of malnutrition than younger children did (thinness in children with CD and overweight in those with UC; *p* = 0.03 and *p* = 0.02, respectively) [12]. This was explained by the widespread and more severe CD in the older age group on the one hand and the different dietary lifestyles and easier access to a high-fat diet in older children with UC on the other hand [12]. Moreover, Kuloglu et al. reported that age of onset (> 10 years) was independently associated with malnutrition in patients with pediatric IBD [21]. However, the studies of Kalantari et al. and Song et al. did not reveal a significant effect of age on patients’ nutritional status, including both the prevalence of thinness and overweight [4, 9].

In terms of the effect of the type of IBD (CD or UC) on nutritional status, the current study showed that despite that weight-for-age and height-for-age at presentation were higher among patients with CD compared to UC, BMI at follow-up was higher among those with UC (*p* < 0.05). Patients with UC were more likely to be overweight than were those with CD (45.6% versus 27.4%, respectively; *p* = 0.042), who tended to be thinner. Similarly, Kuloglu et al. reported higher prevalence of malnutrition in patients with CD (*p* < 0.001) while overweight is more common in those with UC (*p* < 0.001) [21]. Moreover, in the El Mouzan et al. study, patients with CD were significantly thinner than were those with UC [89/255 (35%) patients versus 28/119 (24%) patients, respectively (*p* = 0.037)] who had greater percentage of overweight (20%) than among those with CD (15%) (*p* = 0.219) [12]. Furthermore, Motil et al. found that children with CD were twice as likely to have growth abnormalities than were those with UC [23]. Inflammation caused by CD involves the whole wall thickness and any part of the gastrointestinal tract while that of UC is limited to the colonic mucosa [11]. Subsequently, CD has a more noteworthy impact on nutritional status than does UC [12]. However, Takaoka et al. showed no significant difference between CD and UC patients although patients with CD were more prone to severe malnutrition than were those with UC [11].

This study revealed that postdiagnosis disease duration is a factor that decrease liability to abnormal nutritional status (*p* < 0.001). Patients who were overweight had a significantly longer postdiagnosis disease duration (*p* = 0.005), which might provide them with time for better healing with medical therapy. Nevertheless, Kalantari et al. showed that disease duration was longer in malnourished adult patients with UC than in those without malnutrition, but these differences were not statistically significant [4].

Our study also revealed that azathioprine intake was higher in patients with abnormal nutritional outcomes and overweight specifically (*p* = 0.026). Although azathioprine itself might not directly affect nutritional status, it is an immunomodulator that can help in disease control and subsequently improve nutritional status by restoring nutrient absorption and reducing energy utilization [29]. However, in contrast, azathioprine can cause gastrointestinal intolerance with nausea, vomiting, and loss of appetite, which might reduce the desire to eat [29]. Moreover, the immunosuppressive effect of these agents might increase the risk of gastrointestinal infections causing diarrhea and abdominal pain and reduce the absorption of nutrients and subsequent malnutrition [29].

The current study found that the disease activity has no significant effect on nutritional outcome. However, inflammatory marker levels, CRP levels specifically, were higher in patients with thinness (*p* = 0.007). Moreover, the presence of perianal disease and hematocrit percentage are significant predictors of abnormal nutritional status (*p* = 0.031 and *p* = 0.034, respectively). Several studies have reported an association between disease activity and abnormal nutritional status [4, 8, 9, 11]. Kuloglu et al. revealed that severe disease activity, high CRP level, and perianal involvement were independently associated with malnutrition in pediatric IBD [21]. This difference might be attributed to the small sample size in our study or the variation in the population characteristics.

EEN is the therapy of choice for patients with active CD and growth impairment when it is acceptable to their family and child [2]. Only eight (6.2%) patients in this study received EEN therapy, while most patients refused EEN therapy due to the taste and palatability of the liquid formula, disruption of eating habits, and social stigma associated with nasogastric tube insertion.

Like any other retrospective studies, this study was limited by incomplete demographic data at presentation and by missing data related to patient compliance with medications. Moreover, despite that our hospital is the main center for diagnosis and treatment of patients with pediatric IBD in Bahrain, this study remains a single center study with a small sample size which can limit the generalizability of its findings. Furthermore, the nutritional assessment in this study involved the use of patient weight, height, and BMI, while other anthropometric indices, such as mid-arm circumference and triceps skinfold, were not available. Weight, height, and BMI are crude measures used to assess malnutrition, but little information about improvements in nutritional status has been provided [10]. It is also better to assess patient body composition, such as fat mass and lean mass, to further indicate nutritional status than anthropometry alone [10, 30]. Moreover, among the anthropometric data, the predictors of stunting were not explored in this study because we recently published an article about linear growth impairment in our cohort [31]. Despite these limitations, this paper is important because it is the first study to address the nutritional status of patients with pediatric IBD in Bahrain. After thoroughly searching the literature, most of the reviewed studies reported nutritional status at one point of time and at presentation. Our study reported nutritional status at two points of time, at presentation and during follow up, which makes the findings of our study unique. The nutritional status was assessed, and the results suggested several predictors of the nutritional outcome. Our study stands out and adds new information to the existing knowledge base. In general, reports on IBD from non-Western countries such as Bahrain are valuable contributions to the global epidemiology of these emerging diseases and can form a foundation for any future studies.

## Conclusions

This study highlights the issue of nutritional outcome and factors affecting it in patients with pediatric IBD. It showed a high prevalence of malnutrition among this group of patients. While thinness was anticipated to be high at presentation, overweightness, or even obesity, was unexpectedly high both at presentation and at follow-up. Children with IBD were more likely to become obese when they grow up to adulthood. Weight-for-age, and height-for-age at presentation were higher among CD compared to UC, but BMI at follow-up was higher among UC patients. Thinness was associated with very early-onset disease, lower weight and BMI at presentation, younger age at follow-up, pediatric age group, lower hematocrit, and higher CRP level while overweight was associated with increased weight and BMI at presentation, longer disease duration, older age, and azathioprine intake. Regarding risk factors of nutritional status, Cox regression showed that Bahraini nationality, CD, postdiagnosis disease duration, height, and BMI at presentation, age at follow-up, clinical manifestations including diarrhea, weight loss, perianal disease, and skin rash, and intake of prednisolone were predictors of abnormal nutritional status. Multinomial logistic regression exhibited that very early-onset IBD, weight, and BMI at presentation, level of hematocrit, and intake of multivitamins were predictors of thinness, however Bahraini nationality, normal delivery, CD, postdiagnosis disease duration, weight, and BMI at presentation, age at follow-up, and history of weight loss were predictors of overweight. Specific nutritional screening and therapeutic measures should be tailored to this group of patients. Further studies on the body composition of patients with pediatric IBD, as well as the impact of compliance with medical therapy on nutritional status, are specifically needed.

### Electronic supplementary material

Below is the link to the electronic supplementary material.

Supplementary Material 1

Supplementary Material 2

## Data Availability

Data will be made available on reasonable request from the first author (Dr Hasan Isa).

## References

[1] Kim S, Koh H. Nutritional aspect of pediatric inflammatory bowel disease: its clinical importance. Korean J Pediatr. 2015;58:363 – 8. PMID: 26576179 10.3345/kjp.2015.10.363. 10.3345/kjp.2015.58.10.363 PMC4644763 26576179

[2] Hartman C, Eliakim R, Shamir R (2009). Nutritional status and nutritional therapy in inflammatory bowel diseases. World J Gastroenterol.

[3] Moeeni V, Day AS. Impact of inflammatory bowel disease upon growth in children and adolescents. ISRN Pediatr. 2011;2011:365712. PMID: 22389775 10.5402/2011/365712. 10.5402/2011/365712 PMC3263571 22389775

[4] Kalantari H, Barekat SM, Maracy MR, Azabakht L, Shahshahan Z. Nutritional status in patients with ulcerative colitis in Isfahan, Iran. Adv Biomed Res. 2014;3:58. PMID: 24627866 10.4103/2277-9175.125812. 10.4103/2277-9175.125812 PMC3950790 24627866

[5] Gasparetto M, Guariso G. Crohn’s disease and growth deficiency in children and adolescents. World J Gastroenterol. 2014;20:13219 – 33. PMID: 25309059 10.3748/wjg.v20.i37.13219. 10.3748/wjg.v20.i37.13219 PMC4188880 25309059

[6] Aurangzeb B, Leach ST, Lemberg DA, Day AS. Assessment of nutritional status and serum leptin in children with inflammatory bowel disease. J Pediatr Gastroenterol Nutr. 2011;52. 10.1097/MPG.0b013e3181f87a95. 536 – 41. PMID: 21407117. 10.1097/MPG.0b013e3181f87a95 21407117

[7] Gurram B, Joeckel R, Stephens M (2012). Nutrition in pediatric inflammatory bowel disease. Pract Gastroenterol.

[8] Benjamin J, Makharia GK, Kalaivani M, Joshi YK (2008). Nutritional status of patients with Crohn’s disease. Indian J Gastroentrol.

[9] Song SM, Kim Y, Oh SH, Kim KM (2014). Nutritional status and growth in Korean children with Crohn’s disease: a single-center study. Gut Liver.

[10] Hill (2014). Update on nutritional status, body composition and growth in paediatric inflammatory disease. World J Gastroenterol.

[11] Takaoka A, Sasaki M, Kurihara M, Iwakawa H, Inoue M, Bamba S (2015). Comparison of energy metabolism and nutritional status of hospitalized patients with Crohn’s disease and those with ulcerative colitis. J Clin Biochem Nutr.

[12] El Mozan MI, Al Edreesi MH, Al-Hussaini AA, Saadah OI, Al Qourain AA, Al Mofarreh MA (2016). Nutritional status of children with inflammatory bowel disease in Saudi Arabia. World J Gastroenterol.

[13] El Mozan MI, Almofarreh MA, Saada OI, Al-Hussaini AA, Al-Saleem KA, Almehaidib AI (2016). Impact of pediatric inflammatory bowel disease on linear growth: data from national cohort study in Saudi Arabia. Saudi J Gastroenterol.

[14] IBD Working Group of the European Society for Paediatric Gastroenterology (2005). Hepatology and Nutrition. Inflammatory bowel disease in children and adolescents: recommendations for diagnosis–the Porto criteria. J Pediatr Gastroenterol Nutr.

[15] Bousvaros A, Antonioli DA, Colletti RB, Dubinsky MC, Glickman JN, Gold BD (2007). Differentiating ulcerative colitis from Crohn disease in children and young adults: report of a working group of the North American Society for Pediatric Gastroenterology, Hepatology, and Nutrition and the Crohn’s and Colitis Foundation of America. J Pediatr Gastroenterol Nutr.

[16] WHO Multicentre Growth Reference Study Group. WHO Child Growth Standards: Length/height-for-age, weight-for-age, weight-for-length, weight-for-height, and body mass index-for-age: Methods and development [Internet]. Geneva: World Health Organization. 2006 (312 pages). https://www.who.int/tools/child-growth-standards. Accessed November 16, 2023.

[17] De Onis M, Onyango AW, Porghi E, Siyam A, Nishida C, Siekmann J (2007). Development of a WHO growth references for school-aged children and adolescents. Bull World Health Organ.

[18] Ministry of Health in Bahrain. Growth indicators - children up to five years of age screened in health centers. (2019). https://www.moh.gov.bh/Content/Files/Publications/statistics/HS2019/PDF/CH-08-Primaryhealthcare_2019.pdf. Accessed 18 January 2024.

[19] Sabt AA, Al-Hajeri ME, Haji EA, Al Sharbati W, Ajlan BY, Nassar LM (2019). School screening program in Kingdom of Bahrain: obesity and overweight outcome. J Bahrain Med Soc.

[20] Ministry of Health, World Health Organization. Information & eGovernment Authority. Bahrain National Health Survey 2018. https://www.iga.gov.bh/Media/Agencies/Bahrain%20National%20Health%20Survey%202018%20English.pdf. Accessed 19 January 2024.

[21] Kuloglu Z, Çetin F, Urgancı N, Önal Z, Sarı S, Yüksekkaya H (2022). Nutritional characteristic of children with inflammatory bowel disease in the nationwide inflammatory bowel disease registry from the Mediterranean region. Eur J Clin Nutr.

[22] Filippi J, Al-Jaouni R, Wiroth J-B, Hébuterne X, Schneider SM (2006). Nutritional deficiencies in patients with Crohn’s disease in remission. Inflamm Bowel Dis.

[23] Motil KJ, Grand RJ, Davis-Kraft L, Ferlic LL, Smith EO (1993). Growth failure in children with inflammatory bowel disease: a prospective study. Gastroenterology.

[24] Gharib NM, Rasheed P (2009). Anthropometry and body composition of school children in Bahrain. Ann Saudi Med.

[25] Gharib N, Rasheed P (2011). Energy and macronutrient intake and dietary pattern among school children in Bahrain: a cross-sectional study. Nutr J.

[26] Musaiger AO, Bader Z, Al-Roomi K, D’Souza R. Dietary and lifestyle habits amongst adolescents in Bahrain. Food Nutr Res. 2011;55:7122. PMID: 21912533 10.3402/fnr.v55i0.7122. 10.3402/fnr.v55i0.7122 PMC3171216 21912533

[27] El Mouzan M, Alahmadi N, AlSaleeem K, Assiri A, AlSaleem B, Al Sarkhy A (2020). Prevalence of nutritional disorders in Saudi children with inflammatory bowel disease based on the national growth reference. Arab J Gastroenterol.

[28] Sentongo TA, Semeao EJ, Piccoli DA, Stallings VA, Zemel BS (2000). Growth, body composition, and nutritional status in children and adolescents with Crohn’s disease. J Pediatr Gastroenterol Nutr.

[29] Salzmann M, von Graffenried T, Righini-Grunder F, Braegger C, Spalinger J, Schibli S (2022). Drug-related adverse events necessitating treatment discontinuation in pediatric inflammatory bowel disease patients. J Pediatr Gastroenterol Nutr.

[30] Więch P, Binkowska-Bury M, Korczowski B (2017). Body composition as an indicator of the nutritional status in children with newly diagnosed ulcerative colitis and Crohn’s disease- a prospective study. Gastroenterol Rev.

[31] Isa HM, Mohamed MS, Alahmed FA, Mohamed AM (2022). Linear growth impairment in patients with pediatric inflammatory bowel disease. Cureus.

