# Supplementary material for: Analysis and prediction of nutritional outcome of patients with pediatric inflammatory bowel disease from Bahrain
Source: BMC Pediatr. 2024 Apr 24;24:265. doi: 10.1186/s12887-024-04720-3 (PMC11040799; doi:10.1186/s12887-024-04720-3)
Supplement: Supplementary file 1 — Supplementary Material 1 [file 12887_2024_4720_MOESM1_ESM.docx]

| **Supplementary Table 1** Nutritional status of patients with inflammatory bowel disease at presentation in neighboring countries and worldwide. | | | | | | |
| --- | --- | --- | --- | --- | --- | --- |
| **Country** | **Reference** | **n** | **IBD type** | **Age (yr)^a^** | **Thinness %** | **Overweight %** |
| Bahrain | Our study | 130 | Both | 14.2 (12.6-23.2) | 27.3 | 26.3 |
| Saudi Arabia | El Mouzan et al. [5] 2016 | 375 | Both | 0.33-17 | 31 | 16 |
| Iran | Kalantari et al. [8] 2014 | 99 | UC | 38.3±12.4 | 9.1 | 0.0 |
| Turkey | Kuloglu et al. [22] 2022 | 824 | Both | 12.5 (9.2-14.9) | 14.9 | 3.1 |
| India | Benjamin et al. [7] 2008 | 112 | CD | 18-66 | 52.6 | 0.0 |
| Korea | Song et al. [14] 2014 | 71 | CD | <18 | 27 | NR |
| Japan | Takaoka et al. [11] 2015 | 40 | Both | 31±10 to 39±17 | 95.5-100 | NR |
| Australia | Aurangzeb et al. [6] 2011 | 28 | Both | 0.5-16 | 14.3 | 0.0 |
| France | Filippi et al. [23] 2006 | 54 | CD | 39±2 | 29.6 | NR |
| United States | Motil et al. [24] 1993 | 69 | Both | 12.6±3.3 | 20-49 | NR |
| ^a^Age is presented as median (range) or mean ± standard deviation. IBD, inflammatory bowel disease; UC, ulcerative colitis; CD, Crohn’s disease; NR, no record. | | | | | | |
