# Supplementary material for: Analysis and prediction of nutritional outcome of patients with pediatric inflammatory bowel disease from Bahrain
Source: BMC Pediatr. 2024 Apr 24;24:265. doi: 10.1186/s12887-024-04720-3 (PMC11040799; doi:10.1186/s12887-024-04720-3)
Supplement: Supplementary file 2 — Supplementary Material 2 [file 12887_2024_4720_MOESM2_ESM.docx]

| **Supplementary Table 2** Predicted probability of nutritional status for IBD patients using different risk factors | | | | |
| --- | --- | --- | --- | --- |
| Variables | | Thin | Normal | Overweight |
| Sex | Male | 10.71 | 52.38 | 36.90 |
|  | Female | 13.04 | 54.35 | 32.61 |
| Nationality | Bahraini | 13.39 | 48.21 | 38.39 |
|  | Non-Bahraini | 0.0 | 83.33 | 16.67 |
| Type of delivery | Normal delivery | 17.02 | 59.57 | 23.4 |
|  | Caesarian section | 0.0 | 28.57 | 71.43 |
| IBD type | Crohn’s disease | 13.70 | 58.90 | 27.4 |
|  | Ulcerative colitis | 8.77 | 45.61 | 45.61 |
| Mean pre-diagnosis disease duration of 5.34 mon | | 13.45 | 58.56 | 28.0 |
| Mean post-diagnosis disease duration of 7.24 yr | | 11.99 | 53.52 | 34.49 |
| Mean age at presentation of 10.71 yr | | 10.43 | 53.83 | 35.74 |
| Mean age at follow up of 17.95 yr | | 11.10 | 54.36 | 34.54 |
| IBD onset | Very early onset | 31.25 | 31.25 | 37.49 |
|  | Non-very early onset | 8.77 | 56.14 | 35.09 |
| Diarrhea | Yes | 13.95 | 51.16 | 34.88 |
|  | No | 8.82 | 64.71 | 26.47 |
| Recurrent abdominal pain | Yes | 10.53 | 57.89 | 31.58 |
|  | No | 15.91 | 50.0 | 34.09 |
| Weight loss | Yes | 20.0 | 60.0 | 20.0 |
|  | No | 5.0 | 50.0 | 45.0 |
| Pallor | Yes | 12.44 | 55.10 | 32.65 |
|  | No | 12.68 | 54.93 | 32.39 |
| Anorexia | Yes | 13.04 | 58.70 | 28.26 |
|  | No | 12.16 | 52.70 | 35.14 |
| Vomiting | Yes | 16.67 | 58.33 | 25.0 |
|  | No | 10.71 | 53.57 | 35.71 |
| Arthralgia | Yes | 10.0 | 56.67 | 33.3 |
|  | No | 13.33 | 54.44 | 32.22 |
| Perianal disease | Yes | 13.33 | 52.33 | 33.33 |
|  | No | 12.22 | 55.56 | 32.22 |
| Fever | Yes | 9.09 | 59.09 | 31.82 |
|  | No | 13.27 | 54.08 | 32.65 |
| Constipation | Yes | 11.11 | 61.11 | 27.78 |
|  | No | 12.75 | 53.92 | 33.33 |
| Skin rash | Yes | 0.0 | 84.62 | 15.38 |
|  | No | 14.01 | 51.70 | 34.58 |
| Jaundice | Yes | 33.33 | 66.67 | 0.0 |
|  | No | 11.40 | 54.39 | 34.21 |
| Hematemesis | Yes | 20.0 | 60.0 | 20.0 |
|  | No | 12.17 | 54.78 | 33.04 |
| Extraintestinal manifestations | Yes | 6.38 | 61.70 | 31.91 |
|  | No | 16.44 | 50.68 | 32.88 |
| Disease activity | Mild | 10.0 | 63.33 | 26.67 |
|  | Moderate | 15.22 | 50.0 | 34.78 |
|  | Severe | 12.02 | 47.43 | 40.55 |
| Mean birth weight of 3.10 Kg | | 18.70 | 53.05 | 28.26 |
| Mean weight at presentation of 34.20 kg | | 6.26 | 66.94 | 26.80 |
| Mean height at presentation of 141.04 cm | | 9.62 | 56.97 | 33.40 |
| Mean BMI at presentation of 18.29 kg/m^2^ | | 12.78 | 57.09 | 30.13 |
| Mean hematocrit of 30.71 % | | 10.58 | 52.74 | 36.68 |
| Mean ESR of 29.51 mm/h | | 11.55 | 52.57 | 35.88 |
| Mean CRP of 30.66 mg/dL | | 11.08 | 54.25 | 34.67 |
| Prednisolone | Yes | 14.89 | 54.26 | 30.85 |
|  | No | 4.0 | 48.0 | 48.0 |
| Azathioprine | Yes | 17.65 | 50.59 | 31.76 |
|  | No | 0.0 | 58.82 | 41.17 |
| Mesalazine | Yes | 10.96 | 50.68 | 38.36 |
|  | No | 15.22 | 56.52 | 28.26 |
| Biological therapy | Yes | 18.18 | 47.27 | 34.55 |
|  | No | 7.81 | 57.81 | 34.38 |
| Exclusive enteral nutrition | Yes | 25.0 | 75.0 | 0.0 |
|  | No | 10.66 | 51.64 | 37.70 |
| Omeprazole | Yes | 15.12 | 53.49 | 31.40 |
|  | No | 6.06 | 51.52 | 42.42 |
| Folic acid | Yes | 15.29 | 52.94 | 31.76 |
|  | No | 5.88 | 52.94 | 41.18 |
| Iron supplementation | Yes | 14.08 | 50.70 | 35.21 |
|  | No | 10.42 | 56.25 | 33.33 |
| Vitamin D supplementation | Yes | 16.36 | 50.91 | 32.73 |
|  | No | 9.38 | 54.69 | 35.94 |
| Calcium supplementation | Yes | 19.05 | 40.48 | 40.48 |
|  | No | 9.09 | 59.74 | 31.17 |
| Multivitamins | Yes | 24.32 | 51.35 | 24.32 |
|  | No | 7.32 | 53.66 | 39.02 |
| Ursodeoxycholic acid | Yes | 20.0 | 60.0 | 20.0 |
|  | No | 12.28 | 52.63 | 35.09 |
| Fat soluble vitamins | Yes | 25.0 | 50.0 | 25.0 |
|  | No | 12.17 | 53.04 | 34.78 |
| Other medications | Yes | 27.27 | 27.27 | 45.45 |
|  | No | 11.11 | 55.56 | 33.33 |
| Data are presented as percentage. IBD: inflammatory bowel disease; BMI: body mass index; ESR: erythrocyte sedimentation rate; CRP: C-reactive protein. | | | | |
